# Pitfalls in the pharmacotherapy of hypertension

**DOI:** 10.1016/j.clinme.2026.100582

**Published:** 2026-04-07

**Authors:** Ryan J. McNally, Maisarah Amran, Albert Ferro, Carmela Maniero, Stefano Taddei, Luca Faconti

**Affiliations:** a King's Health Partners, Centre for Translational Medicine, King's College London, United Kingdom; b Hypertension Unit, Guy’s and St Thomas NHS Foundation Trust, London, United Kingdom; c School of Cardiovascular and Metabolic Medicine & Sciences, King's College London British Heart Foundation Centre, United Kingdom; d Barts BP of Excellence, Barts NHS Trust, London, United Kingdom; e Department of Clinical and Experimental Medicine, University of Pisa, Italy

**Keywords:** Blood pressure, Hypertension, Pharmacotherapy, Antihypertensive

## Abstract

Despite the widespread availability of effective antihypertensive medications, blood pressure control remains unsatisfactory in a large number of patients, often due to avoidable dosing misconceptions rather than actual treatment resistance. This educational review aims to facilitate continuing medical education and clinical practice by exploring existing challenges in the pharmacological management of hypertension, focusing on the common drugs prescribed to manage hypertension. After reading this article, clinicians should be able recognise non-linear dose–response relationships, select appropriate medications and doses to achieve better blood pressure control, improve compliance alongside managing side effects that have led to premature discontinuation of beneficial medications. The review further explores how pharmacokinetic properties and patient-specific considerations, such as renal function and ethnicity, might influence choice of antihypertensive medications. This article aims to strengthen clinical choice, medication adherence and long-term management of blood pressure.

## Highlights

• BP control remains poor due to pharmacotherapy pitfalls, not just non-adherence.
• ACE inhibitors have a non-linear dose-response; low doses are often inadequate.
• CCB-related ankle oedema reflects efficacy; it can be managed without stopping treatment.
• Thiazide-like diuretics are preferred over thiazide-type for cardiovascular outcomes.
• Chlorthalidone remains effective in CKD and may be preferred in Black patients.

## Introduction

Hypertension remains the leading modifiable risk factor for cardiovascular disease and affects more than 10 million people in the UK.^1^ Although highly effective antihypertensive therapies are widely available, blood pressure (BP) control is still suboptimal, with roughly one-third of treated patients not achieving recommended targets.^1^ This persistent treatment gap matters: uncontrolled hypertension is strongly linked to preventable stroke, myocardial infarction, heart failure, chronic kidney disease and premature mortality. In many cases, inadequate control reflects shortcomings in pharmacotherapy, rather than true non-adherence to treatment resistance. Optimising drug therapy requires more than choosing the ʻright’ class. It also demands an appreciation that agents within the same class are not interchangeable: drugs can differ markedly in potency, duration of action, pharmacokinetics, metabolic effects, drug–drug interactions and tolerability. These differences influence adherence, 24-h BP coverage, and ultimately clinical outcomes.

This article reviews common pitfalls and practical considerations in the pharmacological management of hypertension, with a focus on three cornerstone classes used in initial and combination therapy – ACE inhibitors/angiotensin receptor blockers (ACE-I/ARB), calcium-channel blockers (CCB) and thiazide/thiazide-like diuretics. It highlights where prescribing choices within these classes can meaningfully affect BP control and patient outcomes, and offers a pragmatic framework to support more effective, individualised treatment.

## Drug-class-specific challenges

### ACE inhibitors and ARB

**Fig. 1:**
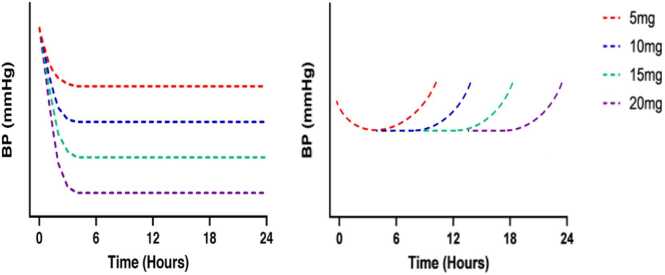
Schematic (illustrative, not to scale) representation of dose–response relationships of antihypertensive drugs. (A) Drugs exhibiting a linear dose–response relationship for blood pressure (BP) reduction and a duration of action sufficient to provide 24 h coverage. For agents with these characteristics, increasing the dose produces proportionate increases in BP lowering and allows dosing to be tailored to the required potency. (B) Drugs exhibiting a non-linear dose–response relationship. For these agents, the magnitude of BP reduction is similar at low and high doses; dose escalation primarily prolongs the duration of action rather than enhancing BP lowering. Adapted from Taddei *et al*.

Current UK hypertension guidelines recommend initiating ACE inhibitors or ARB monotherapy in adults with type 2 diabetes, irrespective of age or ethnic background, and in adults without diabetes who are younger than 55 years and not of Black African or African-Caribbean family origin.^2^ Therapy is generally commenced at a low dose and up-titrated according to clinical response. This strategy is appropriate only for agents with a linear dose–response relationship, in which the BP-lowering effect increases proportionally with the dose administered (Fig. 1a). Such a relationship is typically observed with diuretics, β-adrenoceptor antagonists (β-blockers), β1-selective adrenoceptor antagonists and calcium-channel antagonists (CCB).^3^ In contrast, for drug classes exhibiting a non-linear dose–response curve (Fig. 1b), increasing the dose primarily prolongs the duration of action rather than producing greater BP reduction. This pattern is characteristic of ACE-i and may also apply to some ARBs.

Thus, when titrating ACE inhibitors, clinicians should consider not only the desired magnitude of BP reduction but also the need to maintain consistent 24 h therapeutic coverage. Using an ACE inhibitor at a low dose is often inappropriate, given the pharmacological properties of these drugs, particularly shorter-acting agents that may require twice-daily administration. Importantly, outcome-based randomised controlled trials demonstrating cardiovascular and renal protection with ACE inhibitors and ARBs have almost invariably employed doses at the upper end of the licensed range, underscoring that effective organ protection cannot be assumed at low doses despite apparently adequate BP. To aid clinicians in selecting an appropriate molecule and dose, the British & Irish Hypertension Society (BIHS) has recently issued an expert consensus statement with specific recommendations, including suggested dosing ranges such as lisinopril 10–20 mg once daily and perindopril 4–8 mg once daily.^4^

In many countries, ACE inhibitors are also used less frequently in Black patients due to data suggesting both reduced BP-lowering efficacy compared with other drug classes and higher risk of angioedema. Although evidence, predominantly from the USA, indicates that ACE inhibitors/ARB monotherapy may be less effective than CCBs or diuretics in Black compared with White individuals,^5^, ^6^, ^7^, ^8^ the limited available data on combination therapy suggest a different picture. Evidence from a clinical trial conducted in sub-Saharan Africa indicates that the combination of an ACE inhibitor with a CCB is as effective as a CCB combined with a thiazide diuretic.^9^ Results from the recently completed AIM HY INFORM trial^10^ in the UK will help to further clarify the role of ACE inhibitors as part of combination therapy for hypertension in Black patients.

Regarding angioedema risk, a 2006 systematic review.^11^ based on four US cohorts and randomised trials, along with one multinational study involving US, European, Israeli, Russian and Australian populations, reported a nearly threefold higher incidence of ACE inhibitor-associated angioedema in Black individuals compared to White. Despite the higher relative risk, the absolute risk remained low. Importantly, none of these studies were specifically designed to address this question. In contrast, the large CREOLE randomised controlled trial,^9^ conducted in Black Africans from six sub-Saharan African countries, found an incidence of ACE inhibitor-induced angioedema of 0.7%, a rate comparable to that observed in White populations.^12^

Taken together, the evidence suggests that ACE inhibitors should not be routinely excluded in Black patients, particularly when used as part of combination therapy. When ACE inhibitors are not tolerated or are contraindicated, it is important to consider ARBs as an appropriate alternative; in this respect, dry cough is a well-recognised adverse effect of ACE inhibitors (especially in patients of Asian origin) and often necessitates discontinuation and switching to an ARB. In all cases, clinicians should ensure the use of appropriate doses and long-acting formulations to achieve consistent 24 h BP control, in line with the pharmacokinetic and dose–response characteristics of these drug classes.

### Calcium-channel blockers (CCBs)

CCBs are potent, well-tolerated antihypertensives with robust evidence for cardiovascular protection, especially effective in older adults, those with isolated systolic hypertension, and Black patients. The most common reason for discontinuation of the drug, often reported in studies over 5% of treated subjects, is due to ankle oedema.^13^ Ankle oedema is frequently mistaken for toxicity and should instead be considered as ʻproof of efficacy’. In fact, oedema results from selective precapillary arteriolar dilation without venous compensation, increasing capillary hydrostatic pressure, not fluid retention or organ injury. This effect typically persists despite diuretics and generally reflects pharmacodynamic efficacy rather than harm. Mild forms of oedema can be managed conservatively by maintaining the legs elevated. When oedema is problematic, co-administration of an ACE inhibitor or ARB can reduce its incidence through post-capillary venodilation.^14^ Alternatively, switching to more lipophilic dihydropyridines agent (such as lercanidipine, lacidipine) or non-DHPs CCB (such as diltiazem or verapamil) can improve tolerability while preserving the antihypertensive benefit.^13^

Finally, an often-overlooked approach to reducing CCB-related ankle oedema is gradual dose titration and, in selected cases, nighttime administration (although bedtime dosing may increase nocturia and adversely affect treatment adherence).

Another relatively common reason for discontinuation of CCB is gingival hypertrophy, which is particularly frequent with DHPs such as nifedipine and amlodipine. Gingival hypertrophy is less common with newer CCBs, including lercanidipine, felodipine and non-DHP agents like diltiazem.^15^ Risk is influenced by oral hygiene and local inflammation, and affected patients benefit from switching to lower-risk agents while implementing preventive dental care. Studies show that transitioning to newer CCBs significantly reduces gingival overgrowth, and integrating dental optimisation with careful CCB selection preserves cardiovascular efficacy.^15^ Thus, before discontinuing CCBs due to side effects, clinicians should consider strategies that allow patients to continue benefiting from their BP and cardioprotective effects. In many cases, dose adjustment, switching to a more lipophilic DHP agent, or co-administration with an ACE inhibitor or ARB can help manage adverse effects effectively.^3^

### Thiazide diuretics

Current NICE guidance^2^ explicitly recommends thiazide-like diuretics (indapamide or chlorthalidone) in preference to thiazide-type diuretics (hydrochlorothiazide (HCTZ) or bendroflumethiazide). This reflects both pharmacological differences and the strength of the clinical evidence base. Compared with thiazide-type agents, thiazide-like diuretics generally produce greater BP reduction and appear to confer stronger cardiovascular protection.^16^ Evidence suggests that they are associated with larger reductions in major cardiovascular events, including heart failure and stroke, while adverse-effect rates are broadly similar.

Thiazide diuretics have traditionally been considered less effective in patients with chronic kidney disease (CKD), particularly once eGFR falls below 30 mL/min/1.73 m^2^. Chlortalidone, however, has evidence that it remains effective even in stage 4 CKD, as demonstrated in the CLICK trial,^17^ which showed significant reductions in 24 h systolic BP and albuminuria even in patients already receiving loop diuretics. Whether some of the actions can be explained by its pharmacokinetic properties, which differ from those of other thiazides (highly lipid solubility, extensively accumulates in erythrocytes, and has a long half-life (>40–60 h)) remained to be fully elucidated.

Importantly, chlortalidone also appears particularly effective in Black patients, who tend to have lower plasma renin levels and a more salt-sensitive hypertension phenotype. Outcome data from ALLHAT showed that chlortalidone was superior to ACE inhibitors in preventing cardiovascular events and more effective than CCB in preventing heart failure in Black participants.^18^ These findings, combined with its favourable pharmacokinetic profile, could support selecting chlortalidone when a diuretic is required in patients with CKD or in Black individuals, particularly when achieving durable 24 h BP control is essential.

A practical challenge in the UK is that most single-pill combination antihypertensive formulations contain HCTZ rather than chlorthalidone. Although observational data have reported an association between high cumulative HCTZ exposure and non-melanoma skin cancer, in line with the current Medicines and Healthcare products Regulatory Agency recommendation and an expert consensus document produced by the BIHS,^19^ the decisions of not using HCTZ should not be driven solely by this concern. Furthermore, evidence from the large Veterans Affairs trial demonstrated that HCTZ and chlortalidone provide similar benefits for cardiovascular outcomes, suggesting that when used at truly equivalent doses (particularly in individuals without CKD), the difference between the two agents may be less pronounced.^20^ Nevertheless, chlortalidone’s longer half-life and greater potency remain attractive, especially in Black patients and individuals with kidney impairment.

In summary, optimal hypertension management requires careful selection and dosing within drug classes to maximise efficacy, maintain 24 h efficacy, and minimise adverse effects. Tailoring therapy to individual patient characteristics and pharmacokinetic considerations can help to improve BP control.

## CRediT authorship contribution statement

**Albert Ferro:** Writing – review & editing, Supervision. **Carmela Maniero:** Writing – review & editing, Supervision. **Luca Faconti:** Writing – review & editing, Writing – original draft, Supervision, Conceptualization. **Maisarah Amran:** Writing – review & editing, Writing – original draft. **Ryan J. McNally:** Writing – review & editing, Writing – original draft. **Stefano Taddei:** Writing – review & editing.

## Funding

This research did not receive any specific grant from funding agencies in the public, commercial, or not-for-profit sectors.

## Declaration of competing interest

The authors declare that they have no known competing financial interests or personal relationships that could have appeared to influence the work reported in this paper.
